# Suboptimal Immune Reconstitution among HIV-Infected Saudi Patients following Successful Antiretroviral Treatment

**DOI:** 10.1155/2019/1842106

**Published:** 2019-01-10

**Authors:** Fahad Al-Majid, Zahid Shakoor, Mazin Barry

**Affiliations:** ^1^Infectious Diseases Division, Department of Medicine, King Saud University, King Khalid University Hospital, Riyadh, Saudi Arabia; ^2^Immunology Department, Department of Pathology, King Saud University, King Khalid University Hospital, Riyadh, Saudi Arabia

## Abstract

**Background and Objectives:**

Variations in immune reconstitution following antiretroviral treatment (ART) among HIV patients have previously been observed. This study aims at assessing immune reconstitution after successful ART among HIV-infected Saudi patients.

**Methods:**

This retrospective study of 240 HIV-infected patients was performed between May 2010 and June 2015 in the HIV center at King Saud Hospital, Riyadh. Data were extracted for CD4, CD8 cell, and CD3/HLA-DR counts along with the viral load from patient records before and after four years of successful ART. The inclusion criterion was patients with CD4 reconstitution of either equal to or more than 400 cells/mm^3^ with an undetectable HIV viral load following ART. Based on their presentation, the HIV patients were grouped into early treatment (ET) and delayed treatment (DT) groups with CD4 counts of 200–350 cells/mm^3^ and less than 200 cells/mm^3^, respectively.

**Findings:**

The pretreatment CD8+ counts of median 865 cells/mm^3^ (interquartile range (IQR) 774–1072) in the DT group declined to median 753 cells/mm^3^ (IQR 574–987; *p* < 0.0001). Moreover, there was a decline in CD8 counts from 703 cells/mm^3^ (IQR 655–747) to 620 cells/mm^3^ (IQR 563–645; *p* < 0.04) in the ET group after four years of successful ART. Pretreatment activation marker (CD3/HLA-DR+) expression of median 29% in the DT group declined to 22% and in the ET group from a median of 23% to 19% after treatment. The CD4/CD8 ratio in the DT group increased from 0.14 (IQR 0.09–0.88) to 0.71 (IQR 0.54–0.9) and from 0.42 (IQR 0.35–0.55) to 0.87 (IQR 0.71–0.98) in the ET group.

**Conclusion:**

Immune reconstitution after successful ART among HIV-infected Saudi patients was associated with a CD8 T-cell population expansion with a suboptimal CD4/CD8 ratio and persistent immune activation. Early initiation of ART appears to favorably influence the CD4/CD8 ratio.

## 1. Introduction

Human immunodeficiency virus-1 (HIV-1) infection is characterized by gradual CD4 depletion, CD8 expansion, and immune activation [1]. During the course of illness among patients with human immunodeficiency virus (HIV) infection, CD4+ T-cell counts and viral load are traditionally monitored in order to assess response to therapeutic intervention. Following successful antiretroviral therapy (ART), CD4 counts tend to remain above 200 cells/m^3^ in almost 99.2% of patients, thereby rendering frequent monitoring unnecessary [2]. Experts from the Department of Health and Human Services have issued guidelines that recommend 6–12 months monitoring of all respondents with CD4 counts above the risk for opportunistic infections [3]. Despite the successful restoration of CD4 counts and HIV suppression following ART, immune activation tends to persist, and CD8 counts seldom normalize [4]. There is an emerging consensus that persistent immune activation and inflammation are due to residual HIV replication and microbial translocation that contributes to CD8 expansion [5].

Reversal of the CD4/CD8 ratio is a characteristic feature of HIV infection [6]. ART restore the CD4/CD8 ratio, but the ratio rarely exceeds one, particularly in the event of delayed therapeutic intervention [6–8]. It has been observed that the reversed CD4/CD8 ratio tends to persist among individuals with an undetectable HIV viral load and CD4 T-cell counts of more than 500 cell/ml [9, 10]. Failure to achieve normalization of the CD4/CD8 ratio has been attributed to persistence of high CD8 T-cell counts [4, 11]. Moreover, it has recently been shown that a low CD4/CD8 ratio inversely correlates with the risk of morbidity and mortality [9, 10, 12]. The aim of this study was to assess the immune reconstitution after four years of successful treatment with ART in an Asian population-based cohort of HIV-infected individuals.

## 2. Materials and Methods

This was a retrospective analysis of data extracted from the medical records of 292 patients infected with HIV between May 2010 and June 2015 at the HIV center, Riyadh. Data for CD4, CD8, CD4/CD8, and CD3+/HLA-DR counts and HIV viral load before initiation of ART and four years after successful ART were extracted from the patients' medical record. During the four years of ART, each patient underwent three monthly assessments for cell counts and HIV RNA. All patients included in the study had an undetectable plasma HIV RNA level for at least 2 years prior to inclusion in the study. CD4, CD8, and CD3/HLA-DR assessments were routinely performed by flow cytometry (BD FACSCaliber). HIV RNA quantification was performed using COBAS AmpliPrep COBAS TaqMan HIV-1 test, version 2.0 (CAP/CTM v2.0) (Roche Molecular Systems, Branchburg, NJ, USA) with the lower quantification limit of 20 copies/ml. Attainment of CD4 cell counts of greater than 400 cells/mm^3^ following ART was a criterion for inclusion in the study. Based on the CD4 counts, HIV patients were grouped into early treatment (ET) and delayed treatment (DT) groups. Those with pretreatment CD4 counts of 200–350 cells/mm^3^ were included in the ET group, while those patients with less than 200 cells/mm^3^ were included in the DT group. Noncompliant patients were excluded from the study. All patients were treated with nucleoside reverse transcriptase inhibitor- (NRTI-) containing regimens, combined with either nonnucleoside reverse transcriptase inhibitors (NNRTIs) in 144 (60%) cases, or with protease inhibitors (PIs) in 96 (40%) cases. The normal acceptable laboratory reference ranges for CD4, CD8, and HLA-DR counts were previously established by immunophenotyping Saudi healthy blood donors. The median count for CD4 cells was 615 (range 500–1300) cells/mm^3^ and for CD8 cells was 420 (range 315–716 cells/mm^3^), CD4/CD8 ratio was 1.7 (range 1.2–2.9), and median CD3/HLA-DR expression was 6% (range 3.3–9%). We used one as a cutoff value for a normal CD4/CD8 T-cell ratio [13]. The median reference value for CD3+/HAL-DR+ was 6% (range 3.3%–9.0%).

### 2.1. Statistical Analysis

Data were analyzed using SPSS® computer software, version 20. All categorical data were summarized as numerical and percentage. Numeric data were summarized as median and interquartile range. Comparisons between groups were performed using the Mann–Whitney *U* test. *p* < 0.05 was considered statistically significant.

## 3. Results

**Figure 1:**
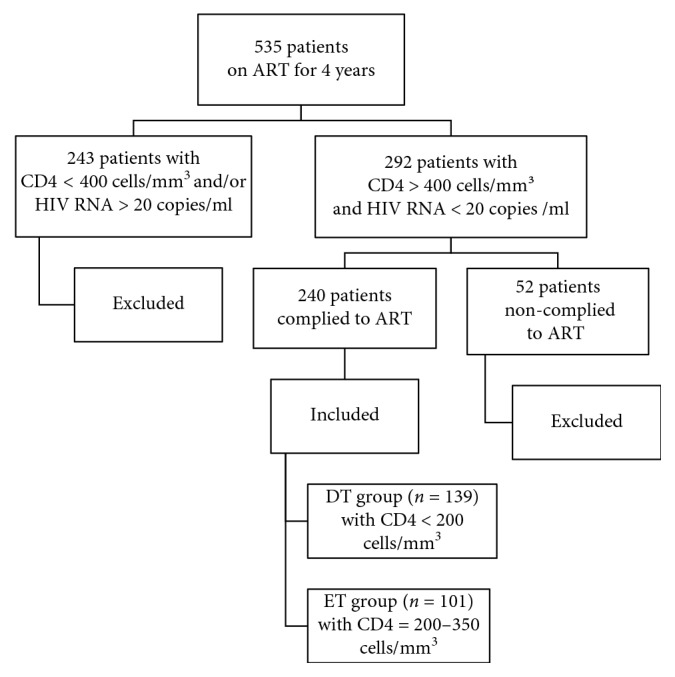
Selection of study population.

During the study period, 535 patients underwent ART. Out of the total, 243 patients were excluded either because of failure to achieve CD4 counts of equal to or more than 400 cells/mm^3^ or due to detectable HIV RNA. In addition, a group of 52 patients was excluded because of noncompliance to ART (Figure 1). Of the 240 patients recruited in the study, 179 (74.5%) were males (median age 33 years; interquartile range (IQR) 18–57) and 61 (25.4%) were females (median age 31; IQR 28–44). The mode of transmission of HIV among 183 (76%) patients was heterosexuality, with 37 (15%) patients acquiring infection via intravenous drug abuse. Ten (4%) patients were homosexuals. However, the mode of infection among the rest of the patients could not be determined (Table 1).

**Figure 2:**
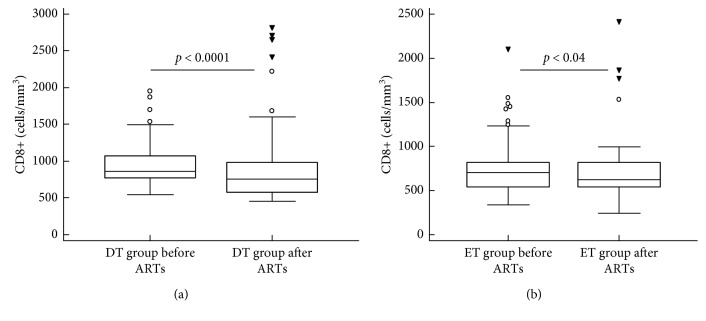
Comparison of CD8 cell reconstitution in delayed and early treatment groups of HIV-infected patients both before and four years after commencement of antiretroviral treatment (ART). ET,  early treatment; DT,  delayed treatment; ART,  antiretroviral treatment.

Alterations in CD4, CD8, and the CD4/CD8 ratio after 4 years of ART are shown in Table 1. HIV viral load declined to <20 copies/ml after 36 weeks of commencement of ART in 95% and 92% of DT and ET groups, respectively. Viral load remained undetectable (<20 copies/ml) for 4 years. The median CD8 T-cell counts among the DT group of 865 cells/mm^3^ (IQR 776.75–1075) declined to a median of 753 cells/mm^3^ (IQR 574–989; *p* < 0.0001) after four years of ART (Figure 2(a)). In the ET group, the median CD8 cell count of 703 cells/mm^3^ (IQR 545–823) decreased to 620 cells/mm^3^ (IQR 537.5–822; *p* < 0.04; Figure 2(b)). Comparative analysis of the reconstitution of CD8 lymphocytes after four years of ART revealed that the CD8 counts among the patients in the DT group (median 753 cells/mm^3^) were higher than in the patients in the ET group (median 620 cells/mm^3^), which remained stable over the years.

**Figure 3:**
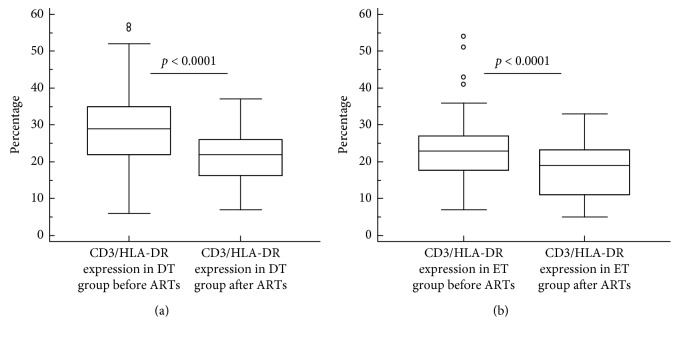
Comparison of HLA-DR expression on CD3+ T lymphocytes between delayed and early treatment groups of HIV-infected patients both before and four years after commencement of antiretroviral treatment. ET, early treatment; DT, delayed treatment; ART, antiretroviral treatment.

Figure 3 compares the data for HLA-DR expression on CD3 lymphocytes before and after four years of ART between the two groups.  Figure 3(a) shows that CD3/HLADR expression in the DT group decreased significantly following four years of treatment with ART from a median of 29% (IQR 22–35) to 22% (IQR 16–26; *p* < 0.0001). A similar reduction in CD3/HLADR expression was also observed among patients in the ET group (median 23%; IRQ 17.5–27 vs. 19% IRQ 11–23.5; *p* < 0.0001; Figure 3(b)). The overall median CD3/HLA-DR expression after four years of ART (22%) remained higher than the normal acceptable median of 6% (range 3.3–9%).

**Figure 4:**
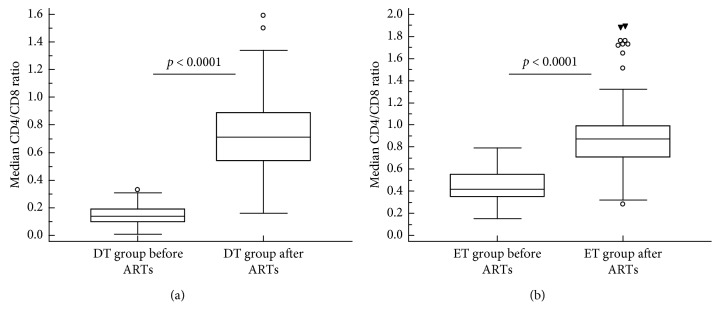
Comparison of CD4/CD8 ratios between delayed and early treatment groups of HIV-infected patients both before and four years after commencement of antiretroviral treatment (ART). ET, early treatment; DT, delayed treatment; ART, antiretroviral treatment.

**Figure 5:**
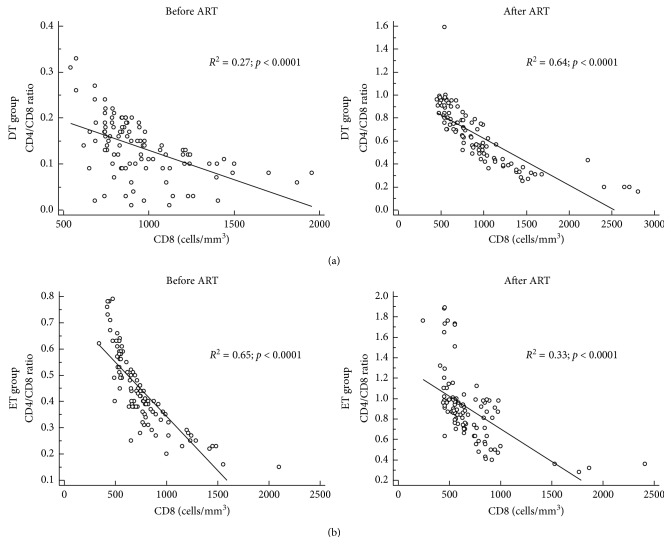
Regression analysis of C4/CD8 ratio and CD8 cell counts before and after antiretroviral treatment (ART) in both DT (delayed treatment) and ET (early treatment) groups of HIV-infected patients.

The CD4/CD8 ratio before commencement of ART in the DT group (0.14 (range 0.10–0.19)) increased to a median of 0.71 (IRQ 0.54–0.89; *p* < 0.0001) four years after treatment (Figure 4(a)). Similarly, the CD4/CD8 ratio in the ET group improved from 0.42 (0.35–0.55) to 0.87 (IRQ 0.71–0.87; *p* < 0.0001) following treatment with ART (Figure 4(b)). Although the reconstitution of the CD4/CD8 ratio remained less than one in both the groups, it was, however, better in the ET group compared to the DT group. This was also evident among 26 (10%) patients who achieved normal CD4/CD8 ratios comprising of 21/26 (80.8%) patients from the ET group compared to 5/26 (19.2%) from the DT group.  Figure 5 shows regression analysis between the CD4/CD8 ratio and CD8 cell counts, where negative correlation was observed between the two variables before and after ART in both the DT and ET groups. Further analysis of the data by multiple regression analysis with the inclusion of data for CD4 counts and HLA-DR expression revealed that negative correlation between the CD4/CD8 ratio persisted (*p* < 0.0001) in both DT and ET groups. Alterations in CD4 counts exhibited a significant positive correlation with the CD4/CD8 ratio (*p* < 0.0001) in both the groups whereas HLA-DR did not correlate with CD4/CD8 ratio, CD4 counts, or CD8 counts in both the groups either before or after ART.

## 4. Discussion

### 4.1. CD8 T-Cell Expansion and HIV Infection

Acute HIV infection causes initial activation and robust expansion of CD8 T cells, in a manner similar to other viral infections. This study has revealed a significant increase in the baseline CD8 T-cell count among Saudi patients infected with HIV. Expansion of the CD8 T-cell count following exposure to HIV is partially related to HIV-specific CD8 T-cell activation [14–17]. HIV-specific CD8 T cells constitute only about 8–10% of the total circulating CD8 T-cell pool that is involved in the partial control of the HIV viremia [17, 18], which fails to undergo complete eradication and tends to persist [19]. Following commencement of ART, HIV-specific CD8 T-cells numbers tend to decrease, although there is a marked elevation in the nonspecific CD8 T-cell population [18]. This phenomenon is referred to as “bystander activation.” The bystander expansion of CD8+ lymphocytes is believed to be induced by inflammatory cytokines such as IFN-*α*, IL-1b, and IL-15 [20].

We observed that following a moderate reduction after initiating ART, the absolute CD8 counts remained consistently elevated over time despite the achievement of normalization of CD4 counts and undetectable viral load in the vast majority of cases. Similar observations have previously been reported in a number of studies [21–25]. Moreover, we also noticed that the ET group had a comparatively less elevation of CD8 counts compared to the DT group after four years of ART. Increased expression of the HLA-DR molecule, considered as an activation marker, was observed on a higher proportion of CD3 T lymphocytes both prior to and after ART in the present study, thereby reflecting persistent immune activation. It is possible that persistently elevated CD8 T-cell counts may be consequent to persistent immune activation driven by residual HIV particles. Early initiation of ART tends to result in lower CD8 counts, which may possibly indicate that the shortening of the duration of exposure to the antigen is associated with a lower degree of immune activation [24, 26].

Experimental studies in mice have revealed that antigen-specific CD4 memory T cells tend to decline whereas CD8 memory and effector T cells remain elevated for life [26]. These observations suggest that differences in the survival rates of memory and effector CD4 and CD8 T lymphocytes may contribute to CD8 T-cell expansion during immune hyperactivation in HIV infection. During chronic HIV-antigenic exposure, the majority of circulating CD8 cells are terminally differentiated CD8 subsets instead of being naïve or memory cells that continue to decline progressively [13, 27]. However, expansion of the CD8 T cell is not limited to HIV infection, as several studies have linked other viral infections such as CMV to immune resilience in the setting of HIV infection [10].

### 4.2. CD4/CD8 Ratio and Immune Dysfunction

During ART, the CD8 T-cell compartment remains expanded among the majority of patients, resulting in a suboptimal CD4/CD8 ratio normalization [28–30]. An inverted CD4/CD8 ratio is a characteristic feature of HIV infection, which is attributed to a CD4 depletion and a CD8 expansion [11], and often remains below one, especially if ARTs are commenced late [6, 7, 11, 18]. In the general population, only 8% of individuals less than 60 years of age and 16%–20% of individuals over age of 60 years are reported to have a CD4/CD8 ratio of less than one [4, 31]. In this study, we observed a low CD4/CD8 ratio among the study cohort, particularly among 139 (58%) individuals with extremely low ratio that might predispose this group to a higher risk of non-AIDS morbidity and mortality [12, 32]. Improvement of the CD4/CD8 ratio was observed in both groups as a result of CD4 T-cell recovery and CD8 T-cell reduction with the ET group exhibiting a significant increase in the ratio compared to the DT group. However, all the infected individuals, even the early-treated ones, continued to display a decreased ratio compared with the reference value. Only 27 (11%) patients could achieve the normalization of a CD4/CD8 ratio of >1 in the present study, and the majority of these patients (74%) belonged to the ET group.

Tinago et al. have reported a gradual improvement in the CD4/CD8 ratio to more than one over a period of fourteen years among 26% of their study population [33]. Moreover, viral load suppression, high baseline CD4, and low baseline CD8 counts have been proposed as the main predictors of reconstitution of the CD4/CD8 ratio over the cutoff value of one [34]. Another study observed a 10.9% rate of ratio normalization that was associated with a higher baseline viral load [31]. The most likely reason for the low percentage of patients attaining the normal CD4/CD8 ratio, in this study, appears to be low CD4+ T cell at the time of commencement of treatment. Initiation of ART at an early stage after infection is likely to attenuate factors known to impair immune reconstitution, such as microbial translocation, immune activation, and limiting the expansion of latent HIV reservoirs [17–21, 35–38]. Furthermore, early commencement of ART has been linked with a relatively rapid increase in the CD4/CD8 ratio, after initiation of ART [34]. Similar observations have shown that early treatment is not only associated with a quick and sustained immune reconstitution but also with attainment of a CD4/CD8 ratio above one among chronically infected HIV patients [36]. A recent report confirmed these findings in a prospective observational study of 353 patients, where early treatment resulted in better normalization of CD4/CD8 ratios compared to patients with deferred treatment [37]. Observations imply that the persistence of the low CD4/CD8 ratio has been associated with senescence of the adaptive immunity [23, 38]. Immunosenescence is characterized by the accumulation of late differentiated memory cells. Data from different cohorts show that an inverted ratio identifies individuals with immune activation, immunosenescence, and higher risk for non-AIDS morbidity and mortality [8]. Recent studies have confirmed an inverse correlation between a low CD4/CD8 ratio and an increased risk of morbidity and mortality [7, 8, 12, 24]. Moreover, an association between the CD4/CD8 ratio and non-AIDS mortality exhibits no association with CD4 counts [24]. Furthermore, a low CD4/CD8 ratio has been proposed to be a marker for age-associated complications including cardiovascular diseases [39]. Existing data support the use of the CD4/CD8 ratio as a simple and reliable surrogate marker for the efficacy of ART and emphasize the need for reversal of persistently elevated CD8 counts. A CD4/CD8 ratio of less than one is considered to be a reliable predictor of mortality in elderly patients [39]. Treated HIV patients with a CD4/CD8 ratio of 0.3–0.4 have been associated with poor prognosis [18]. In addition, it has been observed recently that a low CD4/CD8 ratio of less than 0.5 is the best predictor of non-AIDS cancer [11].

## 5. Conclusion

Following successful ART for four years persistence of CD8 T-cell pool expansion and a low CD4/CD8 ratio among Saudi patients infected with HIV was in conformity with already published data elsewhere. Suboptimal reconstitution of CD4/CD8 ratio, despite optimal reconstitution of CD4 counts, may be attributed to persistently elevated CD8 counts. Early initiation of ART favorably influenced improvement in CD8 T-cell counts and the CD4/CD8 ratio.

## Figures and Tables

**Table 1:** Characteristics of the study population.

Characteristics	DT group CD4 counts <200 cells/mm^3^ (*n*=139)	ET group CD4 counts 200–350 cells/mm^3^ (*n*=101)
Median age in years (IQR)	33 (18–48)	36 (21–51)
Female gender, number (%)	32 (32)	29 (22)
Body mass index	21.4	22.1
CD4, cells/mm^3^, median (IQR)	132 (95.2–155.7)	312 (271–324.2)
CD8, cells/mm^3^, median (IQR)	865 (774–1072)	703 (655–747)
CD4/CD8 ratio, median (IQR)	0.14 (0.10–0.19)	0.42 (0.35–0.55)
CD3/HLA-DR (%)	29	23
HIV RNA log_10_	4.54 (2.82–6.51)	4.9 (2.91–6.9)
Heterosexuals, *n* (%)	110 (79)	67 (66)
Injectable drug abuse, *n* (%)	15 (11)	12 (12)
Homosexual, *n* (%)	3 (2)	1 (0.9)

DT,  delayed treatment; ET,  early treatment; IQR,  interquartile range.

## Data Availability

The data used to support the findings of this study are included within the article.
